# Epstein-Barr Virus Encephalitis in an Immunocompetent Child: A Case Report and Management of Epstein-Barr Virus Encephalitis

**DOI:** 10.1155/2016/7549252

**Published:** 2016-04-26

**Authors:** Gulsen Akkoc, Eda Kepenekli Kadayifci, Ayse Karaaslan, Serkan Atici, Nurhayat Yakut, Sevliya Ocal Demir, Ahmet Soysal, Mustafa Bakir

**Affiliations:** Division of Pediatric Infectious Diseases, Department of Pediatrics, Marmara University School of Medicine, 34890 Istanbul, Turkey

## Abstract

Epstein-Barr virus (EBV) usually causes mild, asymptomatic, and self-limited infections in children and adults; however, it may occasionally lead to severe conditions such as neurological diseases, malignant diseases, hepatic failure, and myocarditis. Epstein-Barr virus-related neurological disorders include meningitis, encephalitis, and cranial or peripheral neuritis, which are mostly seen in immunocompromised patients. The therapeutic modalities for EBV-related severe organ damage including central nervous system manifestations are still uncertain. Herein, we describe a seven-year-old boy with EBV encephalitis who presented with prolonged fever, exudative pharyngitis, reduced consciousness, and neck stiffness. Cranial magnetic resonance imaging showed contrast enhancement in the bilateral insular cortex and the right hypothalamus. The diagnosis was made by EBV-DNA amplification in both the blood and cerebrospinal fluid samples. He was discharged with acyclovir therapy without any sequelae.

## 1. Introduction

Epstein-Barr virus (EBV) infection is common in children and usually resolves spontaneously [1]. The most common clinical manifestations of EBV infection include infectious mononucleosis, prolonged fever, lymphadenopathy, exudative tonsillopharyngitis, otitis media, and diarrhea [1, 2]. Although extremely rare, EBV may also cause central nervous system (CNS) involvement such as demyelinating disease, acute encephalitis, meningitis, meningoencephalitis, myelitis, polyradiculitis, polyradiculomyelitis, cranial or peripheral nerve palsies, and acute cerebellar ataxia [1, 3, 4]. In addition, EBV-related severe organ damages are usually seen in immunocompromised patients [5].

Epstein-Barr virus (EBV) was found as a causative agent in 2 to 5% of viral encephalitis and meningitis cases [1, 6]. In EBV encephalitis, patients may present with fever, headache, stiff neck, altered mental status, irritability, lethargy, and, rarely, a comatose state [1, 6, 7]. Epstein-Barr virus should be considered as a possible causative agent for any child with acute encephalitis, as clinical findings of EBV encephalitis are usually nonspecific [1]. In the diagnosis of EBV encephalitis, EBV antibodies and nucleic acid amplification tests in blood or cerebrospinal fluid and cranial imaging studies can be useful.

Herein, we describe the case of a 7-year-old boy with EBV encephalitis involving the grey matter, bilateral insular cortex, and hypothalamus, who was discharged with acyclovir therapy without any sequelae.

## 2. Case Report

**Figure 1:**
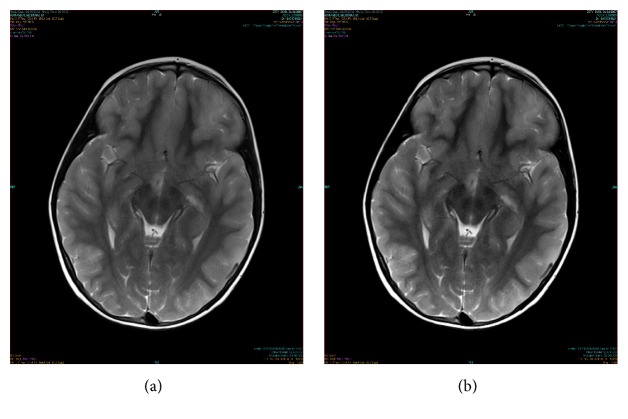
(a, b) Contrast enhancement in the posterior side of the bilateral insular cortex, right hypothalamus, and inferior left frontal cortex consistent with encephalitis.

A 7-year-old boy was admitted to the emergency room with reduced consciousness, incoherent speech, hallucinations, and prolonged fever. He had complaints of nausea, vomiting, and diarrhea for 10 days, and he received oral antibiotics for pharyngitis. The physical examination revealed exudative pharyngitis and neck stiffness. Laboratory analysis results were as follows: hemoglobin: 12.5 g/dL, white blood cell count: 11.3 × 10^9^/L, erythrocyte sedimentation rate: 78 mm/h, and C-reactive protein: 4 g/dL (0–5). Liver transaminases were within normal ranges. Lumbar puncture was performed. Cerebrospinal fluid (CSF) opening pressure was 100 mm/H_2_O. Direct microscopic examination showed 80 erythrocyte/mm^3^ and 20 leukocytes/mm^3^. The CSF protein level was 59 mg/dL and CSF glucose level was 50 g/dL (simultaneous serum glucose level: 130 g/dL). In the initial evaluation, CSF polymerase chain reaction (PCR) analysis for herpes-simplex virus types 1 and 2 and enteroviruses was negative. Vancomycin, ceftriaxone, and acyclovir therapies were initiated empirically, based on the preliminary diagnosis of meningoencephalitis. Magnetic resonance imaging (MRI) showed contrast enhancement in the posterior side of the bilateral insular cortex, right hypothalamus, and inferior left frontal cortex consistent with encephalitis (Figures 1(a) and 1(b)). His level of consciousness worsened, and his Glasgow coma score decreased from 15 to 12 within the first six hours of his admission. He was unresponsive to verbal commands and had resting tremors, rigidity, and hypertonia localized to his right arm. The electroencephalography (EEG) showed no abnormalities. On the third day of admission, a lumbar puncture was reperformed to examine other rare viral causes of encephalitis, autoimmune disorders, and subacute sclerosing panencephalitis, since no clinical improvement was achieved. Real-time quantitative polymerase chain reaction (PCR) of the blood was positive for EBV under the 1500 copies/mL. Real-time quantitative PCR of the CSF was positive for EBV at the level of 1600 copies/mL. Cytomegalovirus, herpes-simplex virus types 1 and 2, parechovirus, echovirus, and* Mycobacterium tuberculosis* PCR analyses produced negative results in CSF. Meanwhile, serum EBV serology was suggestive for primary infection, whereas EBV antiviral capsid antigen (VCA) IgM was positive and EBV anti-VCA Ig G was negative. Serologic testing for West Nile encephalitis, Japanese virus encephalitis, Lyme borreliosis, and measles were all negative in CSF. Vancomycin and ceftriaxone were discontinued, while acyclovir therapy was continued. On the fifth day of admission, his level of consciousness improved. Acyclovir therapy was administered for 14 days. He was investigated for immunodeficiency; however, no significant immunodeficiency was found. His symptoms fully recovered and he was discharged without any sequelae.

**Figure 2:**
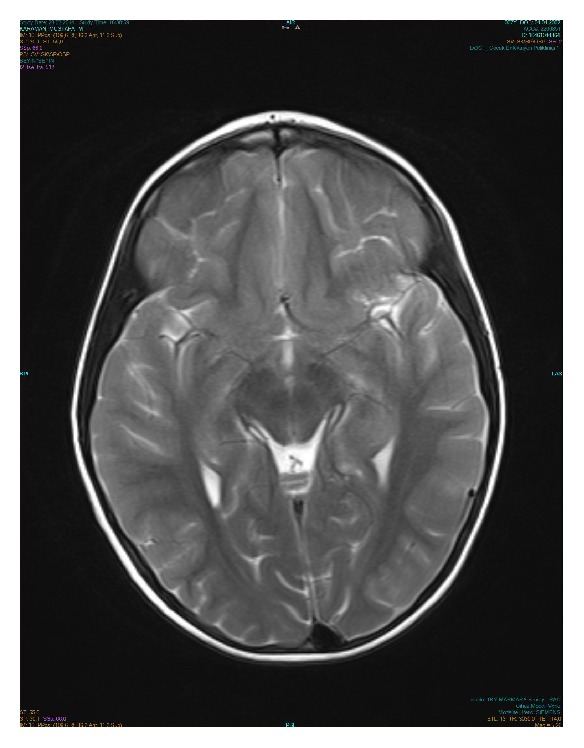
Improvement with a markedly decreased contrast enhancements localized to the insular cortex and frontal lobe, as well as the disappearance of hypothalamic lesions.

In the second month of his follow-up, cranial MRI findings showed improvement with markedly decreased contrast enhancements localized to the insular cortex and frontal lobe and the disappearance of hypothalamic lesions (Figure 2). His physical examination findings were normal and he had no complaints.

## 3. Discussion

Encephalitis, which is a histopathological definition of inflammation of the brain parenchyma, is a severe and fatal disease of the central nervous system [8]. However, EBV rarely causes encephalitis in immunocompromised patients, in particular. In this paper, we describe a case of EBV encephalitis in an immunocompetent child, presenting with typical symptoms of viral encephalitis, such as fever, headache, and altered mental status.

The pathogenesis of EBV encephalitis is still unclear. Neurological complications usually occur concurrently with typical manifestations of infectious mononucleosis; however, they may also present during the resolution phase of infection. The possible mechanisms are described as direct viral invasion to brain parenchyma, the infiltration of cytotoxic T-lymphocytes into the neural tissue, and antibody-antigen complex deposition in neural structures.

In addition, EBV may induce CNS involvement, such as demyelinating disease, acute encephalitis, meningitis, myelitis, polyradiculitis, polyradiculomyelitis, and cranial nerve palsies [3, 4]. Sumaya [1] demonstrated that EBV was a causative agent in 3.6% of cases of 2357 patients living in New York who were diagnosed with meningitis or encephalitis [9].

Although the definite treatment of EBV encephalitis is controversial, previous reports suggested that acyclovir and corticosteroids therapies might be reasonable [10]. Although several reports have demonstrated that antiviral agents including acyclovir, valganciclovir, ganciclovir, and cidofovir have* in vitro* activity against the lytic phase of EBV infections, no antiviral agents are approved for the treatment of EBV infections [9]. Acyclovir may reduce viral replication and nasopharyngeal virus shedding; however, its clinical benefits still remain to be elucidated [11]. In this case, acyclovir therapy was given for 14 days.

Although the prognosis of EBV encephalitis is usually good in the majority of cases (85%), it can be fatal in some patients [12, 13]. In our patient, acyclovir therapy was initiated empirically and it was then continued to manage severe neurological symptoms, even after EBV diagnosis was made. Some reports suggested the use of antivirals for severe EBV infection, which might be beneficial [14]. In a study including 45 patients who had severe manifestations of EBV infection including CNS and peripheral nervous system involvement, thrombocytopenia, aplastic anemia, acute renal failure, and myocarditis, the patients received antiviral therapy; 39 of them had a favorable outcome, while six patients died [14]. In this study, acyclovir was the most commonly given antiviral regimen, as monotherapy in 35 patients [14]. In another study, Bathoorn et al. [15] reported that all patients treated with acyclovir recovered fully except three patients who did not receive antiviral treatment and had persistent symptoms; one of them had a EBV-related malignancy. In our case, we continued antiviral therapy with acyclovir, based on a large number of clinical experiences with this agent in the literature.

Furthermore, cranial MRI is one of the most useful diagnostic modalities in encephalitis cases. It produces a wide range of EBV-related neurological manifestations ranging from a small, localized contrast enhancement to diffuse signal intensity alterations in the white or grey matter and brain atrophy [4, 16, 17]. In some cases, CNS imaging findings can be unremarkable. In the majority of EBV encephalitis cases, MRI findings are transient and usually resolve in a short time period or in several months [7, 18].

In conclusion, EBV encephalitis can be seen in immunocompetent or immunocompromised patients, either in children or adults. Acyclovir therapy may be beneficial; however, further studies are warranted to establish a standard therapeutic approach in the treatment of this patient population.
